# How to improve statistical power in a trial with SCA2 patients using natural history data

**DOI:** 10.1186/s13063-026-09591-w

**Published:** 2026-03-05

**Authors:** Maylis Tran, Pierre-Emmanuel Poulet, Emilien Petit, Alexandra Durr, Thomas Klockgether, Tetsuo Ashizawa, Giulia Coarelli, Sophie Tezenas du Montcel

**Affiliations:** 1 Sorbonne University, Paris Brain Institute (ICM), INSERM, INRIA, CNRS, APHP, Paris, France; 2 German Center for Neurodegenerative Diseases (DZNE), Bonn, Germany; 3 Weill Cornell Medicine at the Houston Methodist Research Institute, Houston, TX USA

**Keywords:** Prediction-powered inference for clinical trials (PPCT), External data, Prognostic covariate adjustment, Prognostic score methodology, Statistical power increase, Digital twins, Natural history data, Clinical trial design

## Abstract

Randomized, double-blind, placebo-controlled trials are the gold standard for evaluating treatment effects, as they minimize biases. However, they raise ethical concerns regarding participant exposure to potentially ineffective interventions. For rare neurodegenerative diseases, these trials are further challenged by the limited number of patients. Our goal is to leverage recent advancements in statistical methodologies to enhance the statistical power of these trials. We aim to test these methods to the ATRIL study, which evaluated the efficacy of riluzole in patients with dominant cerebellar ataxia SCA2. We trained a progression model on natural history data to capture the progression of the disease, using Disease Course Mapping. We explored the prediction-powered inference for clinical trials method (PPCT), which leverages the prognostic scores predicted by this model, to offer an unbiased comparison between the observed progression in treated patients and the predicted progression if they had not been treated. Then, we compared PPCT to the prognostic covariate adjustment and the Hybrid Augmented Inverse Probability Weighting (H-AIPW) methods, which respectively incorporate the prognostic score to the analysis of covariance or to the Augmented Inverse Probability Weighting (AIPW) method. These methods were applied to the ATRIL trial, which included treated and placebo SCA2 patients, using a model trained on patients from US and European cohorts (EUROSCA, SPATAX, CRC-SCA). Disease Course Mapping models accurately forecasted the prognostic scores, namely the predicted one-year SARA score progression, with correlations around 0.15. The treatment effect estimator had a lower variance using the PPCT (0.342), prognostic covariate adjustment (0.344), and H-AIPW (0.348) methods compared to the classical variance (0.407). Notably, PPCT would reduce the sample size by 14.5% (6 patients). Using the PPCT estimator of the average treatment effect, the same variance reported in the ATRIL paper could have been achieved with 39 patients instead of 45. Our study addresses the challenge of statistical power in clinical trials and explores new methodological advancements. Applying the PPCT, prognostic covariate adjustment, or H-AIPW methods helps reduce confidence intervals, enhance statistical power, or decrease the required sample size for the trial by leveraging information from external data. Although the ATRIL’s trial conclusion on riluzole remains unchanged, these methods could have enabled the selection of fewer patients, which is crucial for rare neurodegenerative diseases.

**Supplementary Information:**

The online version contains supplementary material available at 10.1186/s13063-026-09591-w.

## Introduction

Randomized controlled trials (RCTs) are considered the gold standard for assessing treatment effects, as they minimize bias. By the early 1980 s, clinical epidemiologists have regarded RCTs as the highest level of medical evidence [1]. However, these trials raise ethical concerns, as placebo-controlled studies may be ethically problematic for withholding promising new interventions from control groups [2]. Additionally, for rare neurodegenerative diseases, the feasibility of these trials is further complicated by the limited number of eligible patients. Both statistical and clinical significance are crucial when evaluating the effectiveness of a treatment in clinical trials. We consider statistical significance both before and after the trial. Before the trial, this involves determining the required sample size to achieve significance. After the trial, significance can be reassessed by analyzing the results using confidence intervals, which offer deeper insights beyond p-values [3]. Increasing the statistical power of the trial can either reduce the required sample size beforehand or narrow the confidence intervals afterward. By increasing the statistical power of the trial, it becomes possible to address challenges such as the rarity of patients in rare neurodegenerative diseases or the ethical concerns surrounding placebo use, potentially by reducing the number of patients assigned to placebo groups.

Different methodologies exist to enhance trial power and reach significance using statistical methods. For example, Matching Adjusted Indirect Comparisons (MAIC) adjusts for baseline differences between a large dataset of troriluzole-treated subjects and a natural history cohort of ataxia patients [4]. It enhances the estimation of the treatment effect thanks to the large amount of data. However, machine learning is increasingly transforming clinical research across all phases of clinical trials. In the pre-trial phase, it aids in identifying drug targets, while in cohort selection, it enhances patient stratification [5], potentially reducing the sample size needed to observe significant effects. Moreover, machine learning significantly improves data analysis by using existing data to predict outcomes in scenarios that do not yet exist, such as simulating a patient receiving both treatment and placebo [6]. Treated patients can have digital placebo twins, which are virtual representations that resemble the longitudinal characteristics of actual patients [7]. Building on these advancements, the concepts of prediction-powered inference for clinical trials (PPCT) [8], prognostic covariate adjustment [9], and Hybrid Augmented Inverse Probability Weighting (H-AIPW) [10] demonstrate how integrating machine learning predictions from natural history data can further enhance trial methodologies. These methodologies can narrow confidence intervals, improve statistical tests, and reduce sample sizes in experiments.

Spinocerebellar ataxias (SCAs) are a group of rare autosomal dominantly inherited progressive neurological diseases [11]. The most prevalent subtypes are seven SCAs caused by (CAG)n repeat expansions in different genes [12, 13]. Spinocerebellar ataxia type 2 is associated with expansions above 32 CAG repeats in the *ATXN2* gene [14]. The natural history and annual progression rates, assessed using the Scale for the Assessment and Rating of Ataxia (SARA) score, are well-documented for SCA 1, 2, and 3 [15, 16]. To date, there is no curative treatment for spinocerebellar ataxias. In 2022, a new placebo-controlled trial was conducted with riluzole over 12 months (ATRIL), including SCA2 patients only [17]. SCA2 patients were selected since the *ATXN2* expansion is a risk factor for Amyotrophic Lateral Sclerosis (ALS) [18], the mechanism of action of riluzole is established in ALS [19], and motoneuronal involvement is often present in SCA2 [20]. The SARA score indicated a median increase (i.e., worsening) of 0.5 points (IQR –1.5 to 1.5) in the riluzole group versus 0.3 points (–1.0 to 2.5) in the placebo group (*p* = 0.70). No significant difference was observed.

Our objective was to apply recent advancements in statistical methodologies, PPCT, prognostic covariate adjustment, and H-AIPW, to the analysis of the ATRIL clinical trial. Specifically, we sought to illustrate how PPCT, prognostic covariate adjustment, and H-AIPW methods can improve the statistical power of the trial in two significant ways. First, by narrowing the width of confidence intervals on the Average Treatment Effect as measured by the SARA score, thereby empowering the analysis phase. Second, by optimizing the sample size required to achieve statistical significance, which enhances the clinical design. This dual benefit is crucial: it can either improve the accuracy and robustness of trial results or enable a smaller sample size, which reduces the burden of patient recruitment for clinicians and lowers the trial’s overall costs.

## Methods

### Average treatment effect estimator

Let $N$ be the total number of subjects in the clinical trial, ${Y}_{i}$ be the outcome of interest for the patient $i$, capturing the progression of the disease, and ${X}_{i}$ be the covariates at baseline of the patient $i$. The randomized clinical trial presents a treated arm $({T}_{i}=1)$ with $m$ patients and a placebo arm $({T}_{i}=0)$ with $n=N-m$ patients. The value of interest is the Average Treatment Effect (ATE), which is the difference between ${\theta }_{1}={\mathbb{E}}\left({Y}_{i}|{T}_{i}=1\right)$ and ${\theta }_{0}={\mathbb{E}}\left({Y}_{i}|{T}_{i}=0\right)$:

$$ATE= {\mathbb{E}}\left({Y}_{i}|{T}_{i}=1\right)-{\mathbb{E}}({Y}_{i}|{T}_{i}=0)$$

As ${\mathbb{E}}\left({Y}_{i}|{T}_{i}=1\right)$ and ${\mathbb{E}}({Y}_{i}|{T}_{i}=0)$ cannot be known simultaneously for the same patient, we only get estimates of these values, leading to the classic Difference-in-Means estimator:

1 $$\begin{array}{c}{\widehat{ATE}}^{classic}= {\widehat{\theta }}_{1}^{classic}-{\widehat{\theta }}_{0}^{classic}=\frac{1}{m}\sum_{i=1}^{m}{Y}_{i|{T}_{i}=1}-\frac{1}{n}\sum_{i=m+1}^{N}{Y}_{i|{T}_{i}=0}\end{array}$$

with the following variance on the estimator:

2 $$\begin{array}{c}V\left(\widehat{ATE}^{classic}\right)=\frac{\sigma_0^2}n+\frac{\sigma_1^2}m\end{array}$$

where ${\sigma }_{0}$ and ${\sigma }_{1}$ are the standard deviations of the measured outcome for treated and control patients respectively.

Another estimator of the treatment effect is the Augmented Inverse Probability Weighted (AIPW) estimator [21]. When both the outcome regression and propensity score models are correctly specified, which is always true for the propensity model in an RCT, the AIPW estimator achieves the smallest confidence intervals among all consistent and asymptotically normal ATE estimators. These two estimators are computed using only the trial data. They will serve as the baseline methods for comparison against estimators that incorporate external data.

### Prediction-powered inference

PPCT is the adaptation of Prediction-Powered Inference (PPI) to the context of clinical trials. PPI is a linear method that reduces the variance of the average treatment effect (ATE) by leveraging prognostic scores [22]. These scores are predictions of each patient’s outcome, estimated by a machine learning model trained on longitudinal data of natural disease progression. This disease progression model consists in a predictive function$f :X\in \chi \mapsto\mathrm{f}(X)\in {\mathbb{R}}$, which computes the prognostic score$f(X)$, based on the covariates$X$.

**Fig. 1:**
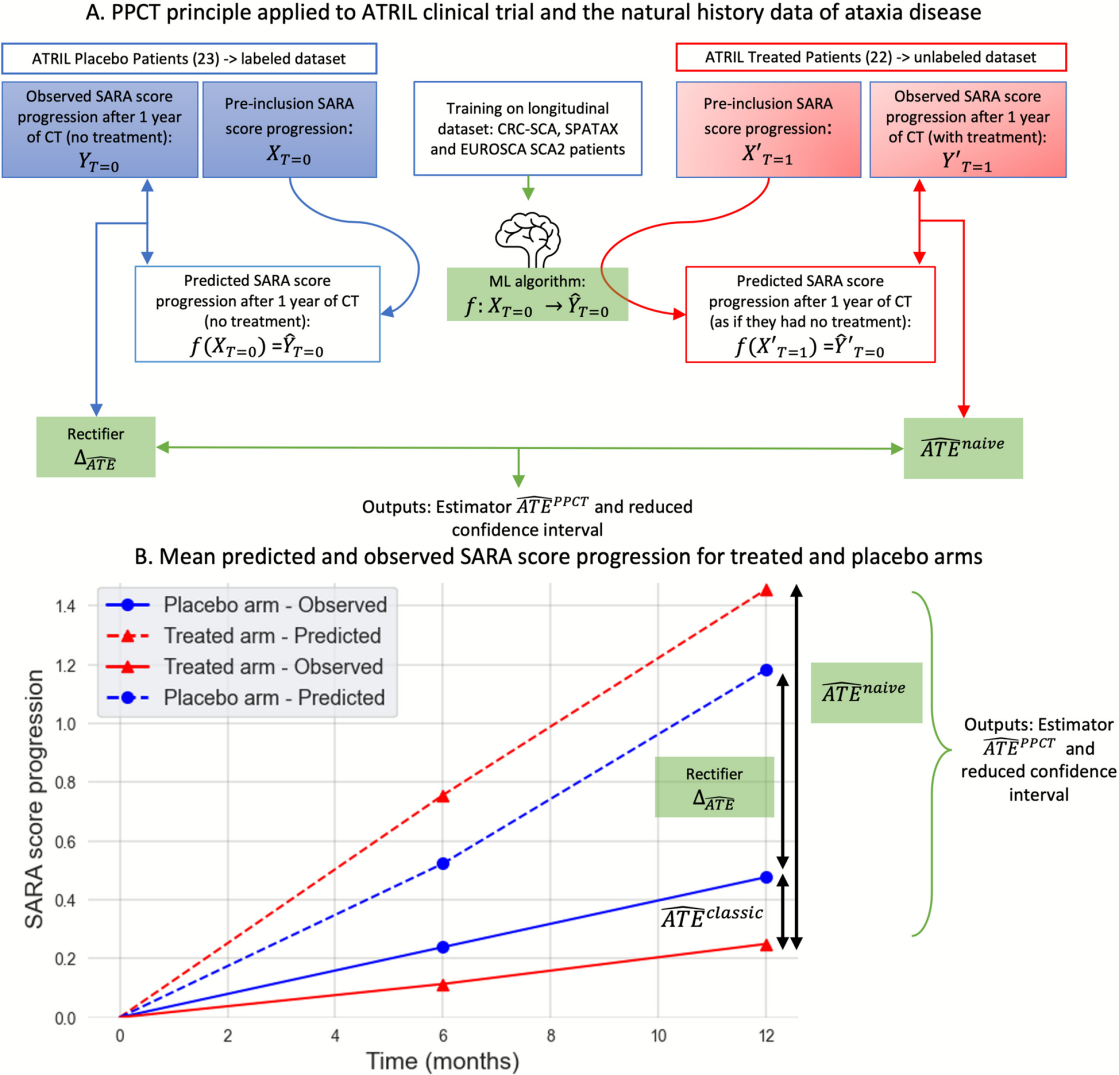
Prediction-Powered Inference for Clinical Trials (PPCT) principle applied to ATRIL Clinical Trial. A progression model—machine learning (ML) algorith—is trained on a longitudinal dataset to capture the natural history disease in untreated patients. This model is then applied to pre-inclusion data from the ATRIL clinical trial to predict off-treatment outcomes and generate a prognostic score (**A**). Comparing the prognostic scores (dashed lines) with the observed SARA score progression over one year (solid line) in the placebo (blue) and treated (red) arms yields the rectifier and the naïve Average Treatment Effect (${\widehat{\mathrm{ATE}}}^{\mathrm{naive}}$), respectively (**B**). By combining the rectifier and the naïve ATE, an unbiased and more accurate estimator of the ATE is obtained

$f\left({X}_{i|{T}_{i}=1}\right)$ is the estimated progression of the disease for the treated patients, considering their individual characteristics, and as if they had not received the treatment (Fig. 1A). Comparing $f\left({X}_{i|{T}_{i}=1}\right)$ with the observed progression of the disease for treated patients lead to a biased estimator (${\widehat{ATE}}^{naive}$), because of the prediction errors of the progression model and the placebo effect (Fig. 1B):

$${\widehat{ATE}}^{naive}= {\widehat{\theta }}_{1}^{classic}-{\widehat{\theta }}_{0}^{naive}= {\widehat{\theta }}_{1}^{classic}- \frac{1}{m}\sum_{i=1}^{m}f({X}_{i|{T}_{i}=1})$$

PPI proposes a rectifier $\widehat{\Delta }$, to unbias the estimator by comparing the real and predicted progression of the disease for the control patients (Fig. 1A):

$${\Delta }_{\widehat{ATE}}=\frac{1}{n}\sum_{i=m+1}^{N}({Y}_{i|{T}_{i}=0}-f({X}_{i|{T}_{i}=0}))$$

The rectifier accounts for both prediction errors and the placebo effect, just as prognostic covariate adjustment considers them during linear adjustment.

This leads to (Fig. 1A):

$${\widehat{ATE}}^{PPI}= {{\widehat{ATE}}^{naive}- \Delta }_{\widehat{ATE}} = \frac{1}{m}\sum_{i=1}^{m}({Y}_{i|{T}_{i}=1}-f({X}_{i|{T}_{i}=1}))- \frac{1}{n}\sum_{i=m+1}^{N}({Y}_{i|{T}_{i}=0}-f({X}_{i|{T}_{i}=0}))$$

PPI +  + is an extension of PPI [23], which proposes a better estimator in which the contributions of the predictions are weighted by $\lambda$:

$${\widehat{ATE}}^{PPI++}= \frac{1}{m}\sum_{i=1}^{m}({Y}_{i|{T}_{i}=1}-\lambda f({X}_{i|{T}_{i}=1}))- \frac{1}{n}\sum_{i=m+1}^{N}({Y}_{i|{T}_{i}=0}-\lambda f({X}_{i|{T}_{i}=0}))$$

Both ${\widehat{ATE}}^{PPI}$ and ${\widehat{ATE}}^{PPI++}$ are unbiased. Since λ is a design parameter, we can optimize it. We choose to minimize $V\left({\widehat{ATE}}^{PPI++}\right)$, which is equivalent to maximizing the power of the statistical test of the clinical trial. This leads to the PPCT estimator, which has the following variance [8]:

3 $$\begin{array}{c}V\left(\widehat{ATE}^{PPCT};\lambda^\ast\right)=\frac{\sigma_0^2}n+\frac{\sigma_1^2}m-\left(\lambda^\ast\right)^2\sigma_f^2\left(\frac1m+\frac1n\right)\end{array}$$

with ${\sigma }_{f}$, the standard deviation of the predictions. The PPCT estimator is no longer unbiased when λ is optimized on the trial data. This bias can be non-negligible in small-sample settings, such as ATRIL trial with 45 patients. The PPCT estimator using λ estimated on the full data can therefore be compared to the version using cross-fitted λ [24].

Since confidence intervals are computed assuming a normal distribution, a smaller variance leads to narrower confidence intervals. From Eqs. (2) and (3), $V\left({\widehat{ATE}}^{PPCT} ;{\lambda }^{*}\right)$ is always lower or equal to $V\left({\widehat{ATE}}^{classic}\right).$ Therefore, PPCT allows to reduce the confidence interval width on the estimator of the average treatment effect.

We define ${\rho }_{0}=\frac{Cov\left(f\left({X}_{i}\right), {Y}_{i|{T}_{i}=0}\right)}{{\sigma }_{f}{\sigma }_{0}}$ and ${\rho }_{1}=\frac{Cov\left(f\left({X}_{i}\right), {Y}_{i|{T}_{i}=1}\right)}{{\sigma }_{f}{\sigma }_{1}}$. In a simplified case where ${\sigma }_{0}={\sigma }_{1}={\sigma }_{f}$ and ${\rho }_{0}={\rho }_{1}$, it is shown that:

4 $$\begin{array}{c}V\left(\widehat{ATE}^{PPCT};\lambda^\ast\right)=\left(1-R^2\right)V\left(\widehat{ATE}^{classic}\right)\end{array}$$

where *R*^2^ is the determination coefficient of the machine learning algorithm. When designing a trial, the determination of the adequate sample size can be improved, as it is proportional to the variance of the estimator, allowing it to be reduced by *R*^2^%. First, PPCT helps to reduce the size of the confidence interval on our treatment effect estimator, potentially allowing us to draw conclusions from trials that might otherwise be inconclusive. Second, it enables the adaptation of trials design by reducing sample sizes, with the same significance that would have been obtained with the classical approach.

Another ATE estimator is the H-AIPW estimator, which combines ideas from PPCT and AIPW estimators. Rather than relying only on outcome regression models fitted on the trial data, H-AIPW incorporates predictions from multiple external data–derived models. Thus, when baseline covariates from the RCT are available and sufficient to yield good predictions, H-AIPW asymptotic variance is no larger than that of the standard trial-only AIPW [10].

### Parallel with prognostic covariate adjustment

The prognostic covariate adjustment method is a linear method, supported by regulatory guidance [25], which uses a prognostic score as a covariate in an analysis of covariate (ANCOVA). As established in [9], the asymptotic variance is expressed as follows:

5 $$\begin{array}{c}V\left(\widehat{ATE}^{ANCOVA}\right)=\frac{\sigma_0^2}{\pi_0}+\frac{\sigma_1^2}{\pi_1}-\left(\frac1{\pi_0\pi_1}\right)\xi_\ast^TV\xi_\ast\end{array}$$

where $${\pi }_{0}={\mathbb{P}}\left({T}_{i}=0\right)$$, $${\pi }_{1}={\mathbb{P}}\left({T}_{i}=1\right)$$, $$V=V\left(f\left(X\right)\right)$$ and $${\xi }_{*}={\pi }_{0}{\rho }_{1}+ {\pi }_{1}{\rho }_{0}$$

From Eqs. (2) and (5), $V\left({\widehat{ATE}}^{ANCOVA} ;{\lambda }^{*}\right)$ is always lower or equal to $V\left({\widehat{ATE}}^{classic}\right)$ asymptotically. This variance has many similarities with the asymptotic variance of PPCT estimator [26]. However, the variance of the PPCT estimator for a sample of size N has an explicit analytic expression as a function of the model parameters compared to ANCOVA. This suggests that the PPCT estimator's analytic variance may be more accurate for small N. In our experiment, we used the “sandwich estimator” which gives the empirical value of the variance, obtained with the ANCOVA.

### Datasets

To apply the PPCT and prognostic covariate adjustment methods, two distinct datasets are required: one consisting of natural history data with longitudinal measures to train the model, and another dataset from a clinical trial to analyze treatment effects.

#### Natural history data

**Fig. 2:**
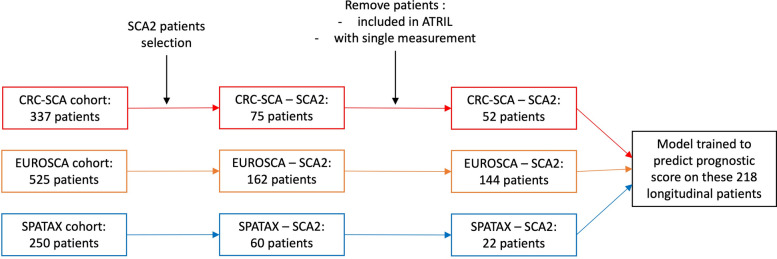
Patient selection process for the longitudinal dataset used to train the longitudinal model. Only SCA2 patients from the identified cohorts were included, as the model is designed to predict the natural history of SCA2 patients within the ATRIL dataset. Patients included in ATRIL were excluded to avoid training on the prediction set, and those with only a single visit were removed. 218 patients with multiple timepoints were selected to train the longitudinal model as a first approach. This leads to a training set of 218 patients and 937 visits in total

Longitudinal data covered up to 14 years of natural disease progression across three cohorts of SCA patients [27]: two European cohorts (EUROSCA [15] and SPATAX [28]) and one from the US (CRC-SCA [29]) with 4.3, 2.2, and 1.1 years of median follow-up respectively (Additional Table 1). Patients with an ATXN2 expansion > 33 CAG repeats were selected for our study, to match ATRIL patients (Fig. 2). Overlapping individuals participating in the ATRIL study were excluded from the natural disease progression training and integrated within the ATRIL dataset. Patients with only one baseline visit were also excluded (Fig. 2). Overall, the 218 patients selected in EUROSCA, SPATAX, and CRC-SCA cohorts had a median of 5, 2, and 3 visits respectively. Natural history data are referred to as the PoolSCA data.

#### ATRIL clinical trial data

A total of 45 patients were selected to participate in the ATRIL clinical trial [17]. Patients were randomized to placebo (23 patients) or riluzole (22 patients, Additional Table 2). Each patient had at least 3 visits: one at baseline (inclusion), one at month 6, and one at month 12. Additionally, natural history data of clinical scores before inclusion were retrospectively retrieved from the medical records. The median number of visits per group was 4 (see Additional Table 2).

### Outcomes

The primary outcome of the ATRIL clinical trial was the progression of the SARA score after one year [16]. The SARA is the primary instrument for assessing the natural history of ataxia. The SARA score ranges from 0 (absence of ataxia) to 40 (the most severe condition). As a secondary outcome, the Inventory of Non-Ataxia Signs (INAS) score was used [30], namely to enrich the prediction model.

### Disease Course Mapping to predict the prognostic scores

**Fig. 3:**
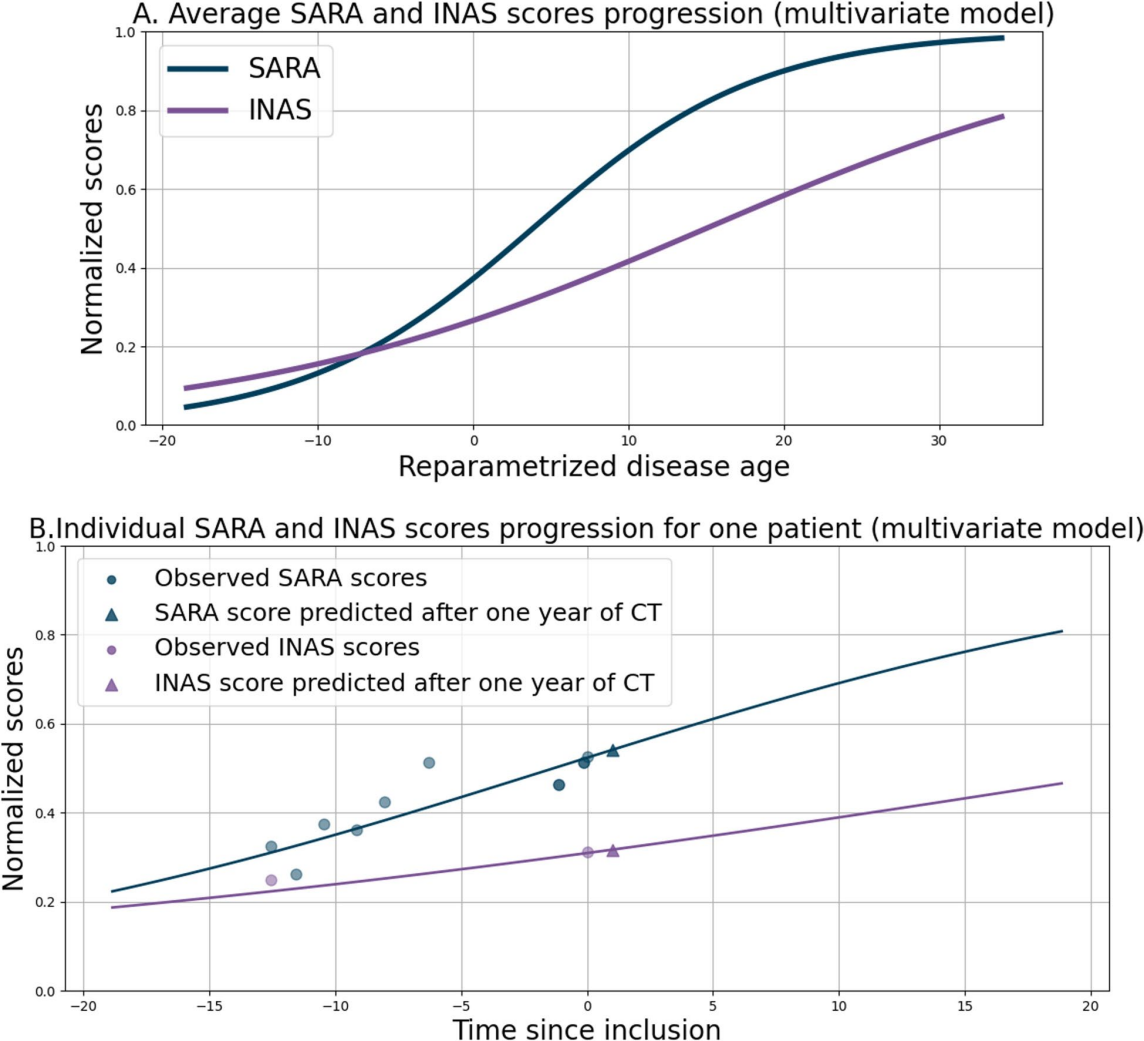
Average (**A**) and individual (**B**) progressions of the SARA and INAS scores with multivariate model. Using Disease Course Mapping with Leaspy library, we can train the longitudinal model to learn the average trajectory of the different features (SARA and INAS) in function of the reparametrized disease age (**A**). To predict the prognostic score of a patient (triangle), we can personalize this average trajectory to the real timepoints of the patients (dots) (**B**). Only the prediction of the SARA score will be used to compute the prognostic score

Several models trained on longitudinal data were used to forecast the natural progression of ataxia, measured with the SARA score progression one year after inclusion, and accounting for what the patients’ progression would have been without the trial. Basic linear regression and linear mixed-effect models were first implemented. Then, a nonlinear Bayesian mixed-effects model, called Disease Course Mapping (DCM) [31, 32], was used to compute the prognostic score. First, longitudinal training data allowed to establish the average progression for the considered outcomes (Fig. 3A). Second, personalization of this progression for individual patients using the observed data before trial inclusion was performed (Fig. 3B). This personalized trajectory led to the estimation of the prognostic score at one year.

The open-source software Leaspy was used to estimate the model parameters from the longitudinal dataset using various disease progression models including univariate, multivariate, and ordinal models. This DCM model is referred to as the “Leaspy” model in the following. In the univariate model, progression of the SARA score over one year was predicted using the total SARA score at each time point. In the multivariate model, we incorporated multiple features (SARA and INAS scores) to improve prediction accuracy. Finally, the ordinal model was trained on baseline SARA items, as they were not reported in the ATRIL study before inclusion. The ordinal model behaves as a multivariate model although it computes the expectation of the sum of the different SARA items [33].

The generalization performance of the models was assessed using the *R*^2^. As the two statistical methods perform linear adjustments, the *R*^2^ score simplifies to the square correlation between the observed and the predicted SARA progression over one year.

One *R*^2^ was computed on the ATRIL data with the prediction model personalized either on baseline data only or on pre-inclusion data in addition to the baseline. In addition, another *R*^2^ was computed as a reference on a subset of the PoolSCA test set using a tenfold cross-validation. In each iteration, the models were trained on nine folds and evaluated on the remaining fold, which was further modified to simulate ATRIL conditions. Specifically, for each patient, the two visits closest to a one-year gap were retained, earlier visits were kept as pre-inclusion data, and any subsequent visits were removed. The *R*^2^ from each fold was then averaged across all ten folds to obtain the final reference R^2^. The combined test folds, referred to as “PoolSCA test set simulating ATRIL,” closely mirror the actual conditions of the clinical trial to avoid overestimating the *R*^2^ and the resulting sample size reduction.

## Results

For the 45 patients included in the ATRIL study, we used Eqs. (1) and (2) to compute the values of the ${\widehat{ATE}}^{classic}$ (${\widehat{ATE}}^{classic}=-\mathrm{0.250}$), its variance (${V(\widehat{ATE}}^{classic})=\mathrm{0.407}$) and the 95% confidence interval of the ${\widehat{ATE}}^{classic}$ ($[-\text{1.501 };\text{ 1.001}]$).

### Models training to obtain the prognostic score

The linear and linear mixed-effects models gave low *R*^2^ (0.032 and 0.087 respectively) (Table 1). The *R*^2^ obtained with univariate, multivariate, and ordinal DCM models on PoolSCA test set simulating ATRIL were higher than the ones obtained in the two linear cases (respectively 0.133, 0.142, and 0.087). Besides, including more variables in the model training improved the accuracy of the model multivariate over the univariate one. The *R*^2^ on ATRIL with pre-inclusions and inclusion visits were 0.145 (univariate) and 0.123 (multivariate), as expected, relatively close to *R*^2^ on the PoolSCA test set simulating ATRIL. When only the baseline visit was used for personalization, *R*^2^ dropped to lower values for the univariate (0.002) and multivariate (0.001) DCM models, while the *R*^2^ with the ordinal model was 0.13.

**Table 1:** Determination coefficients and Average Treatment Effect (ATE) variances for Prognostic Covariate Adjustment (ANCOVA) and PPCT methods

	Linear model	Linear mixed-effect model	Disease Course Mapping		
			Leaspy univariate	Leaspy multivariate	Leaspy ordinal
Determination coefficient (*R*^2^) for the prediction of the PoolSCA data (cross-validation)					
With test set simulating ATRIL	-	-	0.133 [0.050–0.217]	**0.142 [0.058–0.226]**	0.087 [0–0.206]
Determination coefficient (*R*^2^) for the prediction of the ATRIL data					
With baseline only	-	-	0.002	0.001	0.130
With pre-inclusion + baseline	0.032	0.087	0.145	0.123	-
Variances					
$Var (ATE_{PPCT})$	0.395	0.401	0.342	0.351	0.355
$(1-R^{2}) \ast Var(ATE_{classic})$	-	-	0.352	0.349	0.372

For the PoolSCA data, we selected only timepoints similar to the ones in ATRIL and compared the real SARA score progression for one year and the predictions of the model personalized on “PoolSCA test set simulating ATRIL.” A cross-validation process was used to ensure robust evaluation in PoolSCA, as it served as the training dataset, whereas no splitting was necessary for ATRIL dataset, which was used only for predictions. Bold values indicate the best predictive performance (highest R²) among the compared models. For the ATRIL data, we compared the real SARA score progression over one year, and the predictions of the model personalized either on baseline data only or on pre-inclusion data in addition to the baseline. The variances are computed using the sandwich estimator and Eq. (3). As $Var(ATE_{classic})=0.407$, the formula from the Eq. (4) linking *R*^2^ and the variance of the estimator seems to hold empirically. We used the *R*^2^ from PoolSCA data to verify Eq. (4), as this formula is primarily applied to reduce sample size during the clinical trial design phase, where clinical trial datasets like ATRIL are not available

### Outcome regression model specification for AIPW and H-AIPW

The Leaspy multivariate model was built using the SARA and INAS scores. To ensure a fair comparison between PPCT and H-AIPW, we restricted the baseline covariates used in the outcome regression components of both the AIPW and H-AIPW estimators to these same two variables. The outcome regression model was trained using ridge regression to predict SARA score progression based on baseline SARA and INAS scores.

In practice, the outcome regression model achieves better predictive accuracy when using only the baseline SARA score. Therefore, the primary results for both AIPW and H-AIPW are presented under this specification. Because no external trial data with treatment indicators were available, the H-AIPW estimator employed the prognostic models described earlier, which model the outcome without distinguishing treatment arms. This prognostic score served as the outcome regression component in H-AIPW.

### Confidence intervals width reduction

Using the five prognostic scores computed for each patient, we used the PPCT and prognostic covariate adjustment methods to obtain the confidence intervals on the ATE. Given the small sample sizes (22 and 23 patients per arm), in addition to 95% confidence intervals relying on the Central Limit Theorem, we used permutation tests which showed that the test statistic distribution closely matched a normal distribution with the estimator's variance. Thus, only the confidence interval based on the normal distribution is reported.

**Fig. 4:**
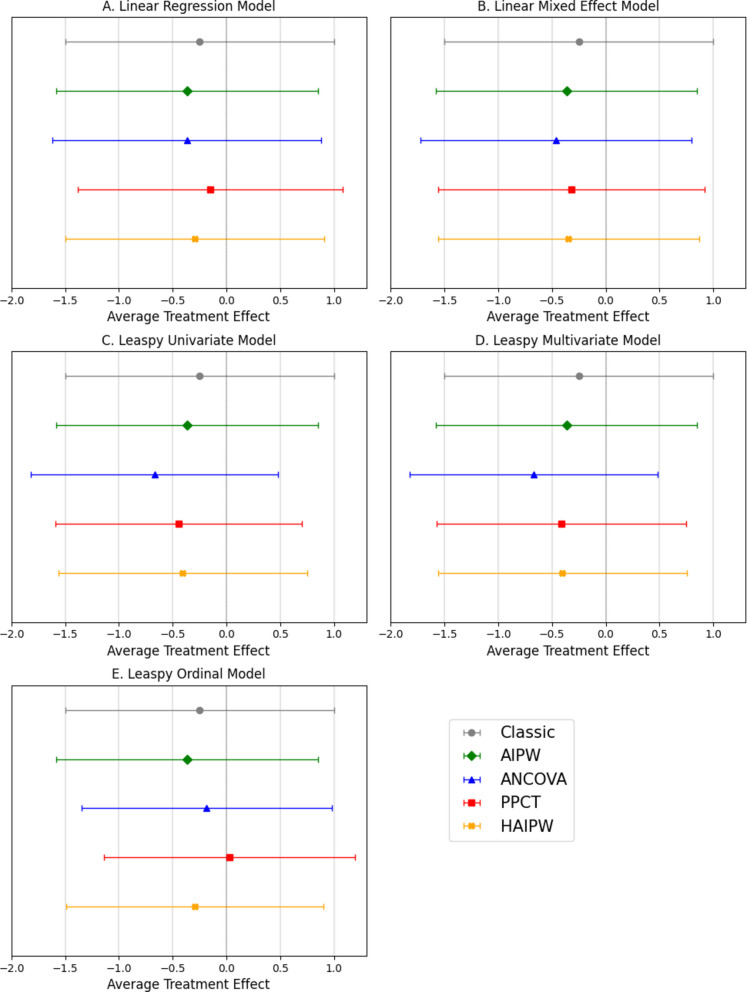
95% confidence intervals on the average treatment effect estimators of ATRIL trial. Linear Model (**A**), Linear Mixed effects model (**B**), or Univariate Disease Course Mapping (Leaspy, **C**), Multivariate Disease Course Mapping (Leaspy, **D**), and Ordinal Disease Course Mapping (Leaspy, **E**) compute the prognostic score. These models are personalized on pre-inclusion and inclusion visits. A companion summary table showing numerical comparisons can be found in the additional material (Additional Table 3)

In line with the decrease of the variance when using DCM modelling to compute the prognostic score, a reduction of the 95% confidence interval width was observed, although the average treatment effect was still not significant (Fig. 4 and Additional Table 3). Using Leaspy univariate prognostic model, prognostic covariate adjustment, PPCT and H-AIPW reduced the confidence-interval width by 8.0%, 8.4%, and 7.6%, respectively, relative to the classical difference-in-means estimator, and by 5.4%, 5.8%, and 4.9% relative to the AIPW estimator. Overall, no single estimator uniformly dominates the others; performance depends on the prognostic model used.

Using PPCT with cross-fitted λ reduced bias, as the PPCT estimator moved closer to the classical ATE while still maintaining a smaller variance when using a sufficiently predictive model like the Leaspy models (Additional Table 4).

### Sample size optimization

**Fig. 5:**
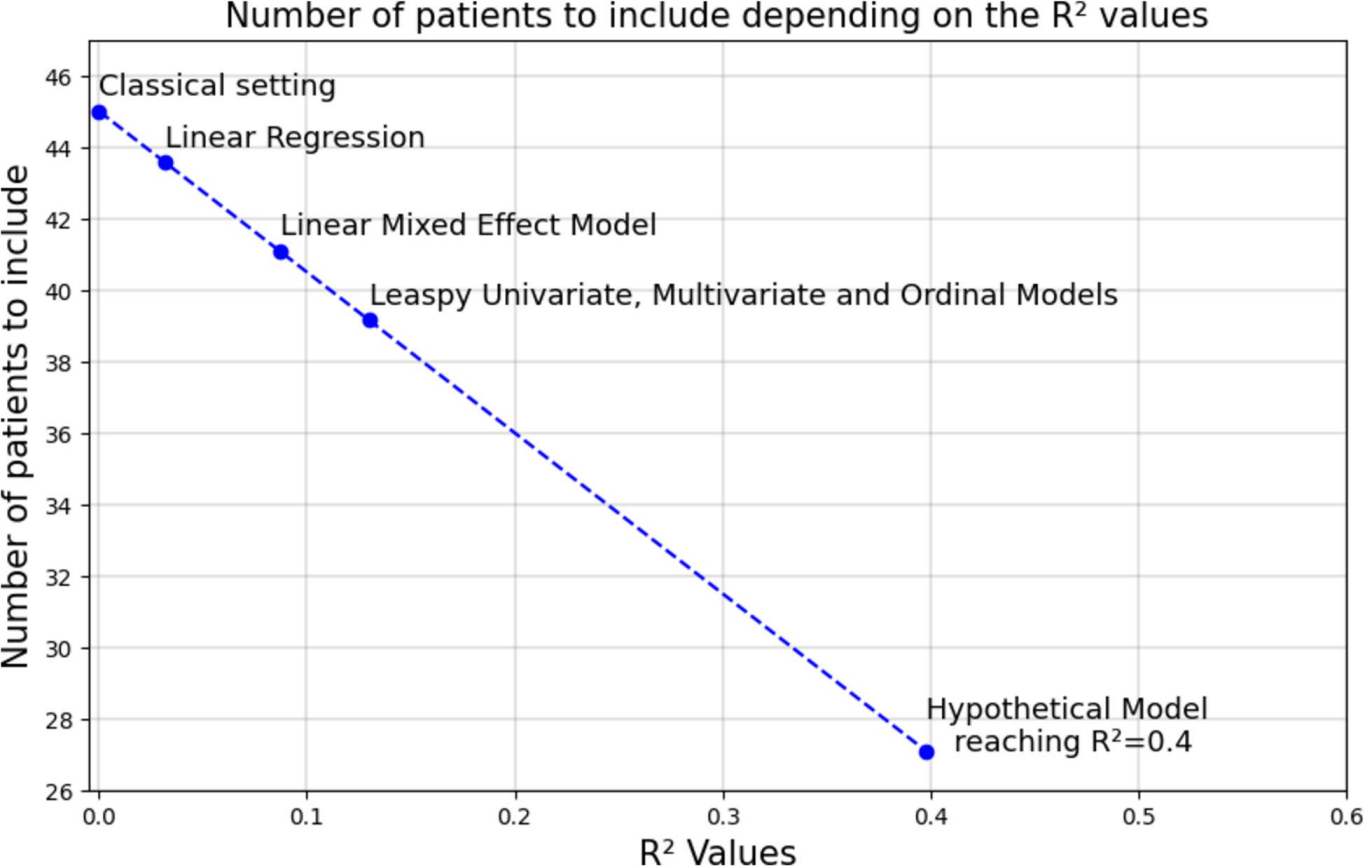
Relation between the required sample size in ATRIL-like clinical trials, according to the *R*^2^ values. The figure shows how the number of patients to include in the clinical trial that mimics the ATRIL trial decreases as the *R*^2^ (determination coefficient) increases, highlighting the efficiency gained by incorporating prognostic scores

The variance obtained using PPCT and prognostic covariate adjustment methods are close but not equal to the ${(1-R}^{2})*Var({ATE}_{classic})$ (Table 1). This rely on the hypotheses beyond Eq. (4) not being fully verified (${\sigma }_{0}={\sigma }_{1}={\sigma }_{f}$ and ${\rho }_{0}={\rho }_{1}$). There is a 14.5% reduction of the sample size in the best case (PPCT estimator using univariate model personalized on pre-inclusion and baseline data). In the ATRIL clinical trial of 45 patients, this represents including 6 patients less and still get the same result obtained in the classic case (Fig. 5).

## Discussion

PPCT, prognostic covariate adjustment and H-AIPW did improve statistical power of the clinical trial by narrowing the confidence intervals, compared to the difference-in-means or AIPW estimators. This reduction occurs because the prognostic score accounts for variability in disease progression that is unrelated to the treatment. It increases the confidence that the observed effects are due to the treatment itself rather than random variation in patient prognoses. As a comparison, both Matching-Adjusted Indirect Comparisons [11] and Bayesian Dynamic Borrowing [34] methods leverage natural history data, using respectively baseline adjustment and Bayesian priors. While these approaches enhance the estimation of treatment effects, they remain biased due to unmeasured confounders. Because the three prognostic score methods are applied in randomized controlled trials, they also incorporate natural history data but without the bias from unmeasured confounders. This holds provided the prognostic score relies only on baseline covariates, the external model is trained independently, standard causal conditions hold, and no post-treatment variables are used. Another potential source of bias in PPCT and H-AIPW arises when the tuning parameter λ is estimated directly on the trial data. This issue can be mitigated by using a cross-fitted estimate of *λ*. In our study, PPCT, prognostic covariate adjustment methods and H-AIPW are leading to the same non-significative conclusions as ATRIL, since the resulting confidence intervals for the ATE include zero. This reinforces the hypothesis that the treatment did not demonstrate efficacy in this trial. Non-significance has also to be considered in the light of high intra-individual variability in SARA score measurements. A study observed the SARA scores assessed by 12 participants over 14 days and found that intra-individual differences between the lowest and highest SARAhome scores ranged from 1 to 5.5 [32].

Prognostic covariate adjustment and PPCT can be used in two different contexts. Firstly, when designing a clinical trial, they can reduce the sample size of *R*^2^% (a priori). Obtaining a meaningful sample size reduction depends therefore on the *R*^2^ and the original or planned trial size. Secondly, when analyzing a trial, they can improve the power of the statistical tests (a posteriori). No matter how accurate the model is, it will always increase the statistical power. However, the better the model predicts the prognostic score, the higher the *R*^2^ is and the more we can reduce the sample size of the trial or the size of the confidence intervals. There are several ways to increase the *R*^2^ value.

First, the longitudinal model trained with Leaspy multivariate is more accurate than simple linear model (+ 10% of *R*^2^). The contribution of DCM to increase the *R*^2^ is therefore considerable, but other machine learning methods, such as Super Learner (SL), can increase the *R*^2^ [35]. SL is an ensemble method that selects the optimal regression algorithm from a set of candidates, which can include both parametric models and data-adaptive algorithms. This approach is particularly effective in reducing bias, especially in cases of severe model misspecification [36]. While SL is powerful for enhancing predictive accuracy across various settings due to its ensemble strategy, it may not capture temporal dynamics as effectively as DCM, which is tailored for analyzing longitudinal data. Using a SL that includes DCM models among its candidate algorithms could enhance accuracy in our context. However, exploring other well suited longitudinal models and implementing the SL is beyond the scope of this study.

Second, in addition to using an appropriate predictive model, having access to diverse data types across multiple timepoints significantly improves the *R*^2^ value. We demonstrated that the more features included in the model, the higher the *R*^2^ becomes. Unfortunately, we could only use two features—SARA and INAS scores—since they were the only ones available in both the training set and the clinical trial. However, other scores like the Unified Huntington’s Disease Rating Scale (UHDRS), the EQ-5D (a measure of health-related quality of life), and the Patient Health Questionnaire (PHQ-9) were available in the longitudinal dataset [27]. If the ATRIL dataset had included these scores along with fluid, imaging, or digital biomarkers, the R^2^ could have been further improved by training the model on more features. Including additional baseline covariates alongside the longitudinal features could improve prognostic accuracy and enable a fairer comparison with H-AIPW, which leverages both covariate information in the AIPW component and longitudinal features in the external outcome regression models. However, this is not feasible with Leaspy, and even if it were, the baseline covariates available in ATRIL have limited predictive value for SARA progression [15]. The performance of H-AIPW could likewise be enhanced with more covariates and if external trial data, not observational data, were available, allowing the outcome regression models to incorporate treatment information.

Third, it is also crucial to note that increasing the number of pre-inclusion timepoints improves prediction accuracy and reduces the width of confidence intervals. One particularly effective technique that leverages pre-experiment data is the CUPED (Controlled-experiment Using Pre-Experiment Data) method [37]. CUPED utilizes pre-existing data to reduce the variability in outcome metrics, thereby improving the precision of estimated treatment effects. The ATRIL dataset, however, presents a limitation in this regard, as a significant portion of patients (26 out of 45) have only one or two timepoints prior to inclusion, since we included early-stage ataxic patients in ATRIL. This sparsity complicates the longitudinal model's ability to accurately tailor predictions. To illustrate this, the relatively low “*R*^2^ on ATRIL with baseline only” obtained indicates that training the models on only one timepoint severely reduces prediction accuracy. With a larger dataset of pre-inclusion timepoints, further improvements in predictive accuracy are expected.

Fourth, the need to use different datasets for training and personalization introduces potential biases due to differences in study design, recruitment and participant characteristics. If the models are trained on data that are not representative of the target population, the predictions might not generalize well for PPCT and prognostic covariate adjustment. This issue is highlighted in a study applying machine learning predictions from a model trained on ADNI to LFAN study, two substantially different datasets [38]. Such discrepancies can undermine the effectiveness of the PPCT approach, particularly when considering differences like age and SARA score at inclusion. To tackle this problem of distribution shift, one approach would be to use propensity scores and reweighting methods [39], which offers a balanced approach to account for covariate differences and align the characteristics of patients across datasets.

These innovations should encourage the development of more refined models, leading to more robust analyses and enhanced statistical power in future clinical trials.

## Conclusions

PPCT, Prognostic Covariate Adjustment, and H-AIPW methods incorporate natural disease progression predictions to reduce confidence intervals, enhance statistical power, and decrease the required sample size for the trial, all while maintaining result validity. Enhancing statistical power in clinical trials is at stake nowadays, due to the challenges surrounding RCTs and the recruitment of patients with rare neurodegenerative diseases.

## Supplementary Information

Additional file 1. Additional Table 1. Longitudinal cohort’s inclusion criteria and follow-up characteristics. Data are expressed as median (IQR). Additional Table 2. Clinical and genetic characteristics at baseline for patients of ATRIL trial. Data are expressed as median (IQR). Additional Table 3. Summary of various Average Treatment Effect (ATE) estimators, using different models computing the prognostic score. Classic difference-in-means, Augmented Inverse Probability Weighted (AIPW), prognostic covariate adjustment (ANCOVA), Prediction-Powered-Inference for Clinical Trials (PPCT), and Hybrid Augmented Inverse Probability Weighted (H-AIPW) estimators are computed. In bold, the smallest values for the variance of the ATE, and the smallest CI width. Additional Table 4. Summary of PPCT analysis using different model to compute the prognostic score and cross-fitting to estimate λ*. The bold cells correspond to the PPCT estimator lying closer to the classical ATE, indicating that they are less biased than the estimators where λ is learned from the same data used to compute the PPCT estimator. The variance is estimated via bootstrap. The PPCT estimator that uses cross-fitting to estimate λ* consistently exhibits higher variance than the PPCT estimator without cross-fitting. However, when the model is sufficiently well specified (e.g., using Leaspy), the cross-fitted PPCT estimator still has lower variance than the classical ATE estimator (0.407)

## Data Availability

The Proposed model implementation is available on GitHub on the open-source package leaspy (v1.5.0): https://github.com/aramis-lab/leaspy, in Python (License BSD 3-Clause). The code for data simulation and experiments is available on a GitLab repository: https://gitlab.com/maylistran01/atril_power_improvement. Note that to run all the same experiments, access to ATRIL and PoolSCA datasets will be required. EUROSCA data are available on the Critical-Path Institute website (https://portal.rdca.c-path.org/), and CRC-SCA data will be uploaded as well in 2024. To request access to SPATAX or ATRIL data and submit a research proposal, please send a request to alexandra.durr@icm­institute.org.

## Abbreviations

SCA2: Spinocerebellar ataxia type 2
SCAs: Spinocerebellar ataxias
PPCT: Prediction-powered inference for clinical trials
H-AIPW: Hybrid Augmented Inverse Probability Weighting
AIPW: Augmented Inverse Probability Weighting
SARA: Scale for the Assessment and Rating of Ataxia
RCTs: Randomized controlled trials
MAIC: Matching-Adjusted Indirect Comparison
ATE: Average treatment effect
ANCOVA: Analysis of covariate
PoolSCA: Natural history data (EUROSCA, SPATAX and CRC-SCA cohorts)
INAS: Inventory of Non-Ataxia Signs
DCM: Disease Course Mapping
PHQ-9: Patient Health Questionnaire
UHDRS: Unified Huntington’s Disease Rating Scale
CUPED: Controlled experiment Using Pre-Experiment Data
