# Supplementary material for: How to improve statistical power in a trial with SCA2 patients using natural history data
Source: Trials. 2026 Mar 5;27:297. doi: 10.1186/s13063-026-09591-w (PMC13072621; doi:10.1186/s13063-026-09591-w)
Supplement: Supplementary file 1 — Additional file 1. Additional Table 1. Longitudinal cohort’s inclusion criteria and follow-up characteristics. Data are expressed as median (IQR). Additional Table 2. Clinical and genetic characteristics at baseline for patients of ATRIL trial. Data are expressed as median (IQR). Additional Table 3. Summary of various Average Treatment Effect (ATE) estimators, using different models computing the prognostic score. Classic difference-in-means, Augmented Inverse Probability Weighted (AIPW), prognostic covariate adjustment (ANCOVA), Prediction-Powered-Inference for Clinical Trials (PPCT), and Hybrid Augmented Inverse Probability Weighted (H-AIPW) estimators are computed. In bold, the smallest values for the variance of the ATE, and the smallest CI width. Additional Table 4. Summary of PPCT analysis using different model to compute the prognostic score and cross-fitting to estimate λ*. The bold cells correspond to the PPCT estimator lying closer to the classical ATE, indicating that they are less biased than the estimators where λ is learned from the same data used to compute the PPCT estimator. The variance is estimated via bootstrap. The PPCT estimator that uses cross-fitting to estimate λ* consistently exhibits higher variance than the PPCT estimator without cross-fitting. However, when the model is sufficiently well specified (e.g., using Leaspy), the cross-fitted PPCT estimator still has lower variance than the classical ATE estimator (0.407) [file 13063_2026_9591_MOESM1_ESM.docx]

**Additional material**

|  | **CRC-SCA** | **EUROSCA** | **SPATAX** |
| --- | --- | --- | --- |
| Study population | Patients with SCA 1, 2, 3 and 6 | Patients with SCA 1, 2, 3 and 6 | Patients with SCA 1, 2, 3 and 6 |
| Ataxia | Presence of symptomatic ataxic disease | Progressive, otherwise unexplained ataxia | - |
| Number of SCA2 patients | 75 (25%) | 162 (55%) | 60 (20%) |
| **SCA2 patients only** | | | |
| Initial SARA score (max value of 40) | 16 (12-20) | 14 (10-19) | 12 (9-13) |
| Age at inclusion | 51 (42-60) | 48 (37-54) | 40 (36-54) |
| Number of visits | 3.0 (2.0-4.0) | 5.0 (4.0-6.0) | 2.0 (0.0-3.0) |
| Follow-up time (years) | 1.1 (0.6-1.6) | 4.3 (3.0-6.5) | 2.2 (1.4-4.4) |

***Additional Table 1. Longitudinal cohort’s inclusion criteria and follow-up characteristics.*** *Data are expressed as median (IQR).*

|  | **Riluzole (n=22)** | **Placebo (n=23)** |
| --- | --- | --- |
| **Sex** | Women : 8 (36%) Men : 14 (64%) | Women : 15 (65%) Men : 8 (35%) |
| **Age at baseline** | 42 (36-57) | 49 (40-56) |
| **Age at onset** | 32 (25-43) | 38 (29-44) |
| **Disease duration** | 11 (5-12) | 11 (6-18) |
| **Repeat length of expanded alleles** | 39 (39-41) | 39 (38-40) |
| **SARA score at baseline (max value of 40)** | 15.3 (10.0-20.5) | 12.5 (9.0-16.0) |
| **INAS count at baseline** | 4 (3-6) | 4 (3-5) |
| **Number of visits** | 4.0 (3.3-5.8) | 4.0 (3.0-5.8) |
| **Number of pre-treatment visits** | 2.0 (1.3-3.8) | 2.0 (1.0-3.8) |
| **Follow-up time (years)** | 2.4 (1.0-3.8) | 1.6 (1.1-2.1) |

***Additional Table 2. Clinical and genetic characteristics at baseline for patients of ATRIL trial*.** *Data are expressed as median (IQR).*

|  | **Classic** | **AIPW** | **Linear Model** | | | **Linear Mixed-Effect Model** | | | **Leaspy Univariate** | | | **Leaspy Multivariate** | | | **Leaspy Ordinal** | | |
| --- | --- | --- | --- | --- | --- | --- | --- | --- | --- | --- | --- | --- | --- | --- | --- | --- | --- |
|  |  |  | **ANCOVA** | **PPCT** | **H-AIPW** | **ANCOVA** | **PPCT** | **H-AIPW** | **ANCOVA** | **PPCT** | **H-AIPW** | **ANCOVA** | **PPCT** | **H-AIPW** | **ANCOVA** | **PPCT** | **H-AIPW** |
| **ATE estimator** | -0.250 | -0.364 | -0.367 | -0.151 | -0.294 | -0.465 | -0.319 | -0.346 | -0.667 | -0.444 | -0.407 | -0.670 | -0.414 | -0.402 | -0.186 | 0.029 | -0.295 |
| **Variance of the ATE** | 0.407 | 0.385 | 0.406 | 0.395 | **0.376** | 0.413 | 0.401 | **0.384** | 0.344 | **0.342** | 0.348 | **0.347** | 0.351 | 0.350 | **0.352** | 0.355 | 0.374 |
| **95% CI Lower Bound** | -1.501 | -1.580 | -1.616 | -1.382 | -1.496 | -1.725 | -1.560 | -1.561 | -1.818 | -1.590 | -1.563 | -1.824 | -1.575 | -1.561 | -1.350 | -1.140 | -1.494 |
| **95% CI Upper Bound** | 1.001 | 0.852 | 0.882 | 1.080 | 0.908 | 0.795 | 0.922 | 0.869 | 0.483 | 0.702 | 0.749 | 0.485 | 0.746 | 0.757 | 0.978 | 1.197 | 0.903 |
| **CI width** | 2.502 | 2.432 | 2.498 | 2.462 | **2.403** | 2.519 | 2.482 | **2.430** | 2.301 | **2.292** | 2.312 | **2.309** | 2.321 | 2.318 | **2.327** | 2.337 | 2.397 |

***Additional Table 3.*** ***Summary of various Average Treatment Effect (ATE) estimators, using different models computing the prognostic score****. Classic difference-in-means, Augmented Inverse Probability Weighted (AIPW), prognostic covariate adjustment (ANCOVA), Prediction-Powered-Inference for Clinical Trials (PPCT), and Hybrid Augmented Inverse Probability Weighted (H-AIPW) estimators are computed. In bold, the smallest values for the variance of the ATE, and the smallest CI width.*

|  | **Classic** | **Linear Model** | **Linear Mixed-Effect Model** | **Leaspy Univariate** | **Leaspy Multivariate** | **Leaspy Ordinal** |
| --- | --- | --- | --- | --- | --- | --- |
| **ATE estimator** | -0.250 | -0.114 | **-0.248** | **-0.412** | **-0.385** | **-0.014** |
| ***~~Variance of the ATE~~*** | *~~0.407~~* | *~~0.341~~* | *~~0.411~~* | *~~0.361~~* | *~~0.368~~* | *~~0.337~~* |
| **Bootstrap variance of the ATE** |  | 0.438 | 0.492 | 0.367 | 0.373 | 0.401 |

***Additional Table 4.*** ***Summary of PPCT analysis using different model to compute the prognostic score and cross-fitting to estimate*** $\boldsymbol{\lambda}^{\boldsymbol{*}}$**.** *The bold cells correspond to the PPCT estimator lying closer to the classical ATE, indicating that they are less biased than the estimators where* $\lambda$ *is learned from the same data used to compute the PPCT estimator.* *The variance is estimated via bootstrap. The PPCT estimator that uses cross-fitting to estimate* $\lambda^{*}$*consistently exhibits higher variance than the PPCT estimator without cross-fitting. However, when the model is sufficiently well specified (e.g., using Leaspy), the cross-fitted PPCT estimator still has lower variance than the classical ATE estimator (0.407).*
